# A New Method of Testing the Dynamic Deformation of Metals

**DOI:** 10.3390/ma14123317

**Published:** 2021-06-15

**Authors:** Jacek Pawlicki, Zbigniew Stanik, Adam Płachta, Andrzej Kubik

**Affiliations:** 1 Department of Railway Transport, Faculty of Transport and Aviation Engineering, Silesian University of Technology, Krasińskiego 8, 40-019 Katowice, Poland; 2 Department of Road Transport, Faculty of Transport and Aviation Engineering, Silesian University of Technology, Krasińskiego 8, 40-019 Katowice, Poland; zbigniew.stanik@polsl.pl; 3 Department of Production Engineering, Faculty of Materials Engineering, Silesian University of Technology, Krasińskiego 8, 40-019 Katowice, Poland; adam.plachta@polsl.pl

**Keywords:** flywheel machine, dynamic deformation, strain rate, tension, impact strength

## Abstract

This paper presents the characteristics of a modernized rotary hammer equipped with a new measuring system based on strain gauges for recording short-term signals. The stand makes it possible to carry out dynamic tensile and bending tests in the range of linear speed of the exciting element from 5 to 40 m/s. Initial tests of dynamic deformation and structural studies in the form of fractures carried out on a representative group of metallic materials allowed determining the correlation “strain rate–strain structure”. The proposed new methodology of dynamic materials testing is an original achievement of the authors and may be an effective tool for assessing the properties of construction materials under conditions of dynamic deformation. In practice, the test results can be used to design the structures of energy-consuming elements of vehicles and aircraft load-bearing elements subjected to dynamic loads. Having an extensive database of results from dynamic tests will allow verifying the correctness of calculations of the structure with the use of the finite element method.

## 1. Introduction

Currently, the possibility of conducting research and determining material characteristics under conditions of high strain rates is limited by the availability of test equipment, the ambiguity of the applied methods, calculation procedures and the complexity of the phenomena accompanying these deformation methods [1,2,3,4]. Thus, designers and technologists do not have information on the behavior of materials under conditions of high strain rates. The need to have this information results from the need for new materials characterized by an unprecedented set of strength and functional properties, in order to meet the increasingly stringent requirements for the construction of means of transport and the rigorous conditions for conducting acceptance tests of materials and structural elements [1,2]. Crash tests are commonly used in the automotive industry, and the aviation industry has advanced approval procedures for new technical solutions [3,4].

The priority of the safety of users of means of transport poses more and more challenges to designers and technologists regarding the mechanical and functional properties of construction materials, and acceptance tests of new materials and construction elements are increasingly demanding. The most commonly used methods of assessing the plasticity of materials are tensile, compression, torsion and impact tests, as well as model upsetting and rolling tests [5,6]. Used to determine them, inter alia, standard testing machines allow tensile tests to be carried out with a strain rate of up to 0.1 s^−1^.

In the torsional plastometer test, the maximum strain rates are in the order of 10 s^−1^. Even the very modern and widely used test system in materials research “Gleeble” allows carrying out tests with a strain rate in the range from 0.0001 to 100 s^−1^ [7,8,9]. The range of deformation velocities defined as “dynamic deformation of the material” is very wide. For this reason, it is impossible to carry out the entire range of tests on one device and using one type of measuring apparatus. Some of these devices are standard laboratory equipment, and the tests performed on them are standardized. This applies, for example, to swing hammers, where the Charpy impact bending test is commonly known and used, and also in the conditions of industrial laboratories. In the case of modified Hopkinson bar systems, where strain rates in the range of 500 s^−1^–10^5^ s^−1^ are obtained, the problem is the availability of the apparatus and the complexity of the procedures for analyzing the results [10,11,12]. Most often, tests are carried out in tests for compressing cylindrical samples because this method of loading is simple and effective. The samples are small in size due to the permissible loads. This is rarely stretching or twisting due to the complexity of the sample. Hopkinson’s modified rod is invaluable in research analyses for higher deformation speed ranges. Unfortunately, measuring systems are complex and extremely sensitive to external interference.

The possibility of testing materials with deformation rates from 10^2^ to 10^4^ s^−1^ is provided by rotary hammers. These devices are characterized by a compact design and an uncomplicated test method. An additional advantage of these devices is the possibility of testing samples made of rods as well as coating materials, sheets and strips. This versatility of application, a large range of deformation rates and a relatively low cost of making test samples encourage the search for the possibility of using these devices in testing materials [13,14,15,16].

The mechanical design of the device is not new. The rotary hammer has been completely upgraded and equipped with a new registration system. Rotary hammers were used in the 1970s. There are few such devices in Europe. Certainly, three devices are also in Poland, and a few are in Germany. There are more of these devices in Germany because they were constructed there. These devices operate on the basis of various measurement systems, which are the result of many years of work of research teams. For example, a rotary hammer operating at the Wrocław University of Technology has a measuring system similar to Hopkinson’s rod measuring system [17]. It is equipped with a rod on which tensiometry in the bridge layout is placed in the lower part. Such a long element was intended to prevent the overlap of the so-called reflected wave on the tensile graph, thereby eliminating possible signal interference. However, it caused excessive damping of the measuring signal and a decrease in the level of force value. There is no such drawback in our system. The tensiometric force measuring device shall be fixed at the top of the handle in direct contact with the test. An additional argument in favor of the registration system developed is the comparison of the results with those obtained on the original rotary hammer measuring system based on a piezoelectric sensor. The results are comparable and, for some materials, e.g., steel 0H18N9 (EN 1.4301), differ only in the second digit after the decimal place. Additionally, importantly, the nature of the force flow curve depending on the time F = f (t) is very similar. The reliability of the results obtained must not be in doubt. In a few research centers, teams have the ability to verify their measurement systems.

**Figure 1:**
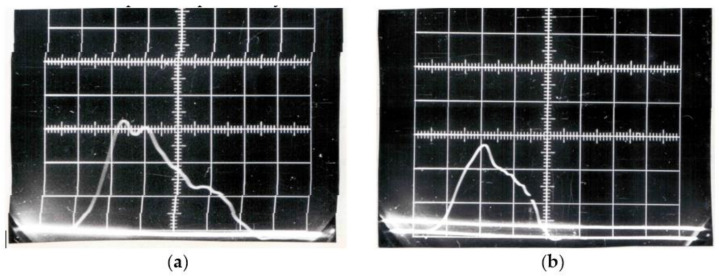
Images of the force pulse oscilloscope screen for hot-tool steel WCL (EN 1.2343 [18]) in the dynamic tensile test for linear speed of the beater v = 10 m/s (**a**) and 20 m/s (**b**).

Figure 1 shows examples of steel yield curves obtained in the original measuring system equipped with a piezoelectric force sensor. These are images of the oscilloscope screen recording a force pulse taken with a camera whose shutter was coupled to the moment the beater in the rotary hammer flywheel was triggered. The system was modern at the time. For understandable reasons, technical progress, the computerization of measuring systems has become useless. Between 1990 and 2009, there were virtually no tests carried out on these devices. The images of the impulse force recorded on the photographic film are from 1986 to 1987, and these are the last studies using this system. The rotary hammer was upgraded and equipped with a new measuring system in 2009. A similar situation occurred in the case of a rotary hammer at the Wrocław University of Technology. Both hammers were upgraded with funds from the same national research project. The development of a methodology and tests verifying the measuring tracks of the system is a long and continuous process, practically unfinished.

The problem of dynamic deformation tests, also carried out on rotary hammers, is the course of the obtained force characteristics and their interpretation. Professor Janusz Klepaczko, who devoted his entire scientific life to the problems of plastic deformation of materials under dynamic loads and the accompanying phenomena, stated that “learning about the plastic properties of metals deformed with high strain rates, of the order of 5 × 10^2^ s^−1^ and higher, is a serious experimental problem” [19]. There are serious doubts as to the correctness of the analysis of the results, especially in the initial stage of deformation. Are the observed diversified waveforms of force characteristics caused by the specificity of the structure and operation of the dynamic testing device, the structure of the grips, the shape of the sample or the ability to identify and filter disturbances? The common use of dynamic tests in industrial solutions is also conditioned by the cost of the tests. Currently, these costs, compared to the standard ones (static tensile test, impact bending test, etc.), are much higher; therefore, despite the existence of rational premises for carrying them out, we do not observe any significant use of the results of such tests in the processes of designing materials and new structures. Obtaining answers to only some of these questions will make it possible to validate the results of dynamic deformation tests and the use of material characteristics in computer simulation programs and in the design of materials and new structures for the automotive and aviation industries.

The rotary hammer is characterized by ease of use, simplicity of the recording system and uncomplicated sample geometry. The method of implementation of the test is similar to a static tensile test and a simple bending test. The authors’ intention is to interest the scientific community in a new research method. The use of dynamic plasticity characteristics in new materials and design processes is insufficient. There are no material characteristics available under high-speed deformation conditions above 500 s^−1^. The purpose of the work is to present the device and the original test methodology, which will certainly work in both scientific and industrial laboratory conditions [20,21,22,23]. The studies present, intentionally, test results for materials representing steels and nonferrous metal alloys, and they want to take an interest in the new method of environmental specialists of the aerospace (PA4, AZ31) and automotive (STEEL DP and TRIP) industries.

## 2. Materials and Methods

**Figure 2:**
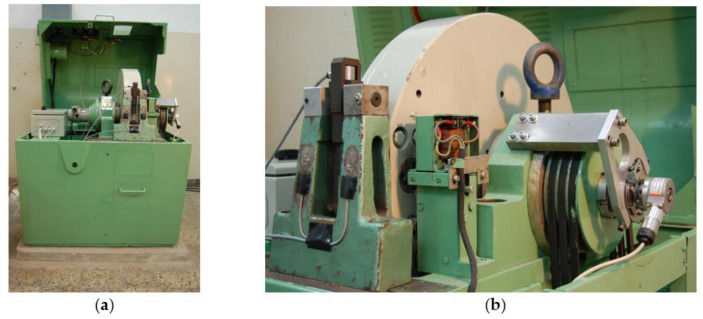
The stand for dynamic tests (**a**); the workspace of the flywheel machine (**b**).

**Figure 3:**
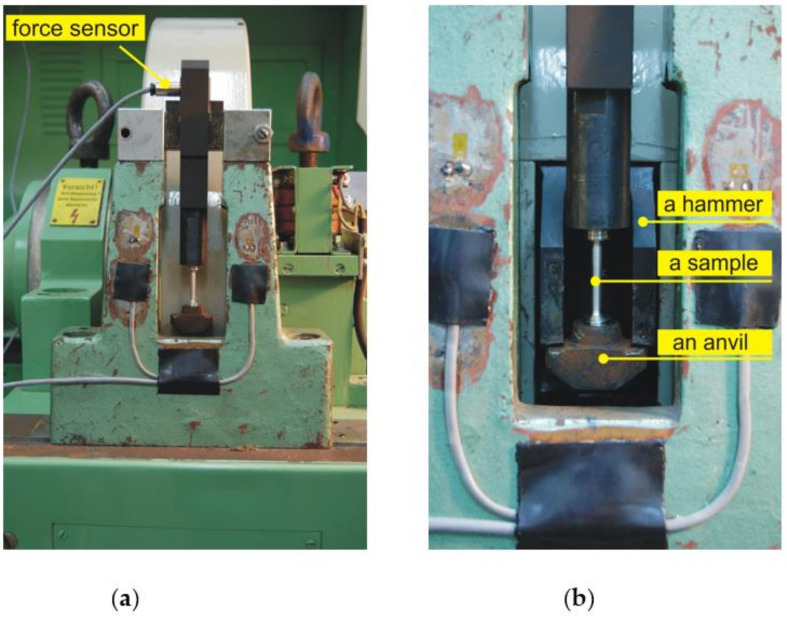
Rotating hammer working space in dynamic tensile test with a tensiometric sensor in the upper handle (**a**); sample with an anvil mounted at the bottom (**b**).

**Figure 4:**
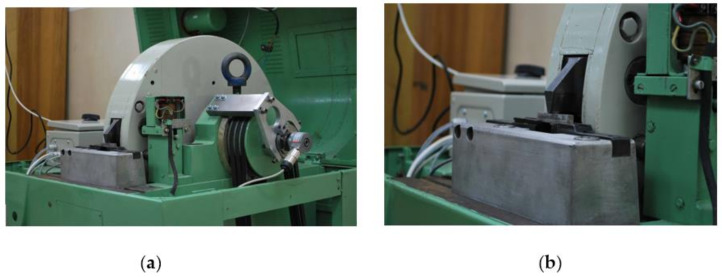
View of the rotary hammer in impact bending tests (**a**); the sample resting on the anvils and the claw in the working space (**b**).

The tests of dynamic deformation were carried out on a rotary hammer of the RSO (rotary impact hammer) type shown in Figure 2 and Figure 3. The device allows for the stretching and bending of samples with a linear impact velocity in the range from 5 to 40 m/s, which corresponds to obtaining a deformation speed in the range from 10^2^ to 10^4^ s^−1^. During the dynamic tensile tests, the sample is connected to the upper grip and is subjected to deformation by hitting the hammer (claw) against the anvil of the lower grip. On the other hand, during impact bending, the sample is broken on the anvil with a hammer claw (Figure 4). The replaceable claw is mounted on a flywheel with a very high moment of inertia. Measurement of force during tensile or bend tests is carried out on the basis of a strain gauge pressure sensor with a nominal range of 25 kN, the design of which was adapted to be installed in the elements of a tripod or anvil, depending on the nature of the test. The linear speed of the hammer in the measuring system was determined by measuring the rotational speed of the hammer flywheel. The speed was measured using an encoder installed at the end of the hammer shaft journal. The Kübler (Villingen-Schwenningen, Germany) 0520 encoder with a resolution of 3600 imp./rpm was used to distinguish the road of about 0.5 mm on the radius of the hammer beater. The linear velocity of the beater is determined by determining the frequency of the rectangular waveform obtained at the encoder output.

The rotary hammer measuring system developed and made for measuring the force (tensile, bending) and linear speed of the beater consists of the following basic components:

- Force sensor and strain gauge;
- Encoder and module f/U for assigning frequency changes to voltage changes;
- Measuring card with 16-bit A/C converters;
- Computer PC.

**Figure 5:**
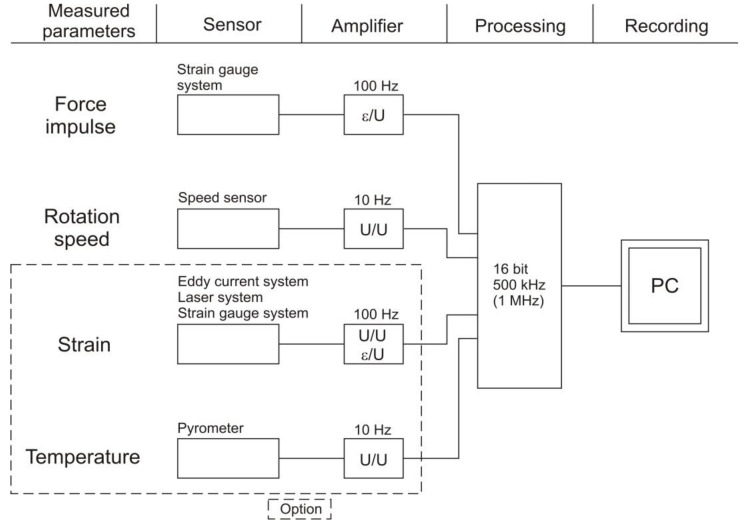
Diagram of the rotary hammer measuring system with the option of expanding the system.

The diagram of the measuring system with optional expandability of the system is shown in Figure 5. For signal conditioning, a measurement card of the PCI-BASE 1000 type was used with a sampling frequency of 500 kHz, allowing for the synchronous reading of both signals, every 2 μs. The Next View 4.2 software by BMC, Germany, was used to control the system and record the signal.

Temperature measurement is an essential issue in the optional scheme of the device due to the dynamic deformation in adiabatic conditions, without heat transfer with the environment. Knowing the temperature increase of the material during deformation will allow a proper assessment of microstructural changes caused by high deformation speed. The tests of dynamic deformation were carried out in tensile and bend tests. The test specimens were made of the following metallic materials: steel with the TRIP effect, steel type DP, magnesium alloys AZ31 and aluminum PA4. These materials were subjected to heat treatment in order to obtain the desired and homogeneous initial structure:

- Steel with the TRIP effect—after hot rolling (last pass 910 °C)—structure: ferrite + bainite + residual austenite;
- DP-type steel—hardening in water in the range of ferrite + austenite (temperature 810 °C)—structure: ferrite + martensite;
- AZ31 magnesium alloy after hot rolling—annealing 350 °C/1 h/air;
- PA4 aluminum alloy—supersaturation from the temperature of 500 °C/1 h/water.

**Figure 6:**
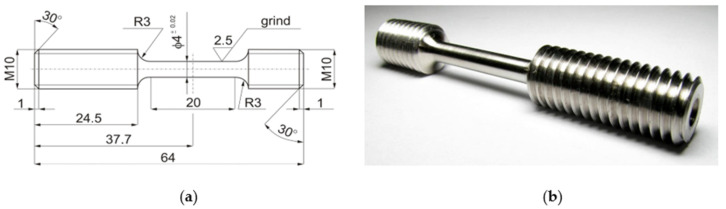
The smooth cylindrical sample (metric unit) for the dynamic tensile test stand for dynamic tests (**a**); the workspace of the flywheel machine (**b**), where R3 is radius 3mm, M10 is diameter 10mm.

In the tensile tests, smooth cylindrical samples with a diameter of 4 mm and a measuring part length of 20 mm were used, threaded on both sides in the grip part (Figure 6). The sample is not covered by the standard and is characteristic only for this device, so it was presented. The breaking of the sample is dependent on the energy of the flywheel with a high mass, which is transmitted when the sample is broken into a beater (claw). The maximum diameter of the measuring part of the sample must not be more than 4 mm (12.56 mm^2^). A larger sample diameter could damage the beater or device. In the case of steels prone to TRIP fortification (in the publication), the sample was broken within the upper measuring range of the strain gauge (25 kN).

In the impact bending tests on a rotational and Charpa-type pendulum, standardized samples were used, made of a bar with a square cross-section of 10 × 10 mm and a length of 55 mm, with a “v” notch with a depth of 2 mm. These tests were carried out for the PA4 aluminum alloy. Tests of dynamic deformation on a rotary hammer were carried out in the range of the hammer linear speed of 5–30 m/s. This corresponds to the obtained deformation velocities in the range of 3 × 10^2^–6.5 × 10^3^ s^−1^. During the tests, the course of the tensile and bending forces was recorded as a function of time and the linear velocity of the hammer located in the flywheel. From the force characteristics and measurements of the sample geometry before and after deformation, the following were determined: deformation velocity, tensile strength, limit deformation and impact strength.

Boundary deformation is defined in logarithmic measure, which is also called a natural measure or Hencky measure. They use the principle of constant volume reduction in the continuous volume of the boundary deformation, which is used in the analysis of plastic deformation processes, determined by the following Equation (1):

(1) $$\varepsilon_{g}=\ln\frac{S_{0}}{S_{1}}=\ln\frac{\frac{\pi d_{0}^{2}}{4}}{\frac{\pi d_{1}^{2}}{4}}=\ln\frac{d_{0}^{2}}{d_{1}^{2}}=\ln{\left(\frac{d_{0}}{d_{1}}\right)}^{2}=2\ln\frac{d_{0}}{d_{1}}$$

where:

*ε_g_*—limit deformation;
*S*_0_—the initial cross-section of the measuring part of the sample;
*S*_1_—cross-section of the sample after deformation at the site of local narrowing (in the neck);
*d*_0_—initial diameter of the sample (mm);
*d*_1_—minimum diameter of the specimen after rupture (mm).

The deformation velocities corresponding to the given hammer linear velocities were calculated from the ratio of the limit deformation εg to the test duration t:

(2) $$\dot{\varepsilon }=\frac{\varepsilon_{g}}{t}$$

where:

$\dot{\varepsilon }$—deformation velocity (s^−1^);
*ε_g_*—limit deformation;
*t*—time (s).

The deformation speed determined from Equation (2) is the average speed. The deformation εg (log measure) is related to the duration of the deformation, from the moment the beater came into contact with the anvil to the breaking of the sample. Determining the deformation speed from the elongation of the sample is difficult due to the deformation of the measuring part. It is impossible to use an extensometer elongation measurement as in a static tensile test because classical extensometers do not allow the elongation to be measured in such short deformation times. In addition, the dynamic deformation course will definitely damage the elements of the extensometer, hence the measurement of deformation from the change in sample cross-section.

The impact strength was determined from Formula (3):

(3) $$U_{t,b}=\frac{L_{u}}{A}$$

where:

*U_t,b_*—toughness in the tensile, bending test (J/cm^2^);
*L_u_*—work of deformation at rupture (J);
*A*—initial section of the sample (cm^2^).

Equation (3) is the standard equation for determining toughness. The number of deformation operations is expressed in J, and the surface area of the sample measuring part is expressed in cm^2^. The deformation work for tensile and bending attempts on rotational dents is determined as the area of the surface under the tensile or bending curve F = f(Δl). The breaking work for attempts to bend on the Charpa pendulum was read directly from the device registration system.

Regardless of the dynamic deformation tests, tensile tests under static conditions on universal a testing machine and bending tests were carried out on a Charpy pendulum hammer with an initial impact energy of 300 J. The same standardized samples were used in the bending tests on pendulum and rotary hammers. The breakthroughs were identified on a Hitachi S-3400 N scanning microscope.

## 3. Results

**Figure 7:**
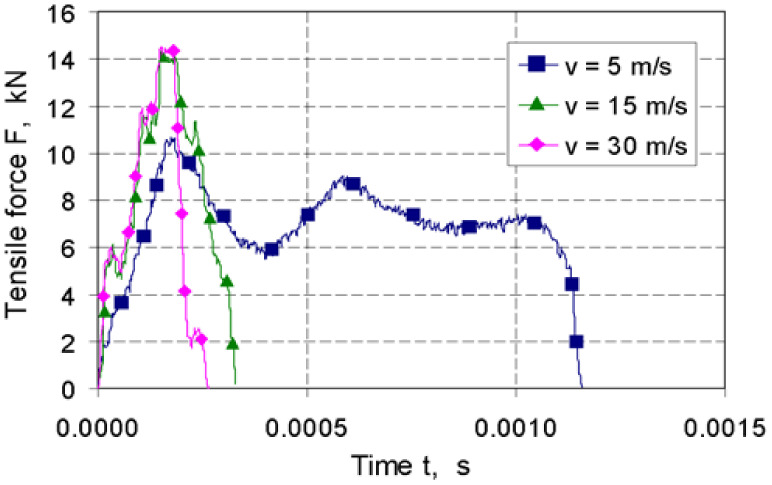
Variation in tensile force with time for TRIP steel at a linear rate of the impact hammer v = 5, 15 and 30 m/s.

During the deformation tests, the course of forces was recorded depending on the time. Examples of the recorded tensile forces for TRIP steel are shown in Figure 7. The recording time of the measurement signal in the system is 10 s, and the capacity of a single data file is 20 MB. 

**Figure 8:**
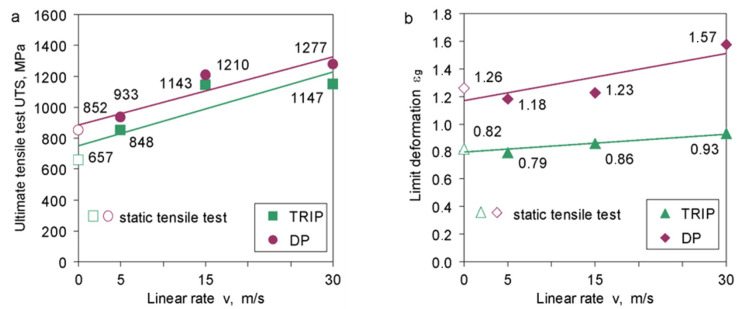
Tensile strength (**a**) and limit of deformation (**b**) as a function of the linear rate of the impact hammer for TRIP and DP.

The dependence of the tensile strength of TRIP and DP steels on the deformation rate is shown in Figure 8a. These steels show particular sensitivity to the deformation rate. The increase in tensile strength is significant compared to the tensile strength determined under static conditions. TRIP steel achieves a tensile strength value of about 1150 MPa for the linear speeds of the hammer v = 15 and 30 m/s. The DP steel achieves the tensile strength of 1200 and 1277 MPa, respectively, for the linear velocities of the hammer v = 15 and 30 m/s. The maximum values of the tensile strength of the tested steels under dynamic deformation conditions are much higher than the tensile strength under static conditions. The limit deformation of TRIP and DP steels, despite the increase in strengthening (tensile strength), stabilizes at a constant level in a certain range of linear velocity, and for linear velocity v = 30 m/s, it shows a slight tendency to increase. This tendency is visible especially for DP steel (Figure 8b).

**Figure 9:**
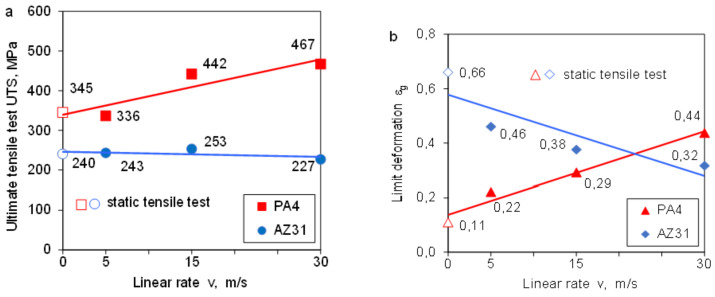
Tensile strength (**a**) and limit of deformation (**b**) as a function of the linear rate of the impact hammer for PA4 and AZ31.

The dependence of the tensile strength of the PA4 and AZ31 alloys on the strain rate is shown in Figure 9a. High sensitivity of the PA4 aluminum alloy to the deformation rate was observed. The strengthening tendency of the PA4 alloy is high for the linear speed of the hammer v = 15 and 30 m/s. The tensile strength of AZ31 magnesium alloy is kept constant. We do not observe any increase in material hardening. A significant and proportional increase in the limit deformation was observed for the PA4 aluminum alloy (Figure 9a). The limit deformation is almost three times greater than that determined in static conditions. The behavior of the magnesium alloy is completely different (Figure 9b).

The limit deformation of AZ31 magnesium alloy clearly decreases with the increase in the deformation velocity, and for the velocity v = 30 m/s, it reaches the value of ε_g_ = 0.32, half lower than that obtained in static conditions (ε_g_ = 0.66). It follows that with the increase in the deformation rate (the linear speed of the ram), the susceptibility of the AZ31 magnesium alloy to plastic deformation decreases. The plasticity reserve of the material is exhausted. 

**Figure 10:**
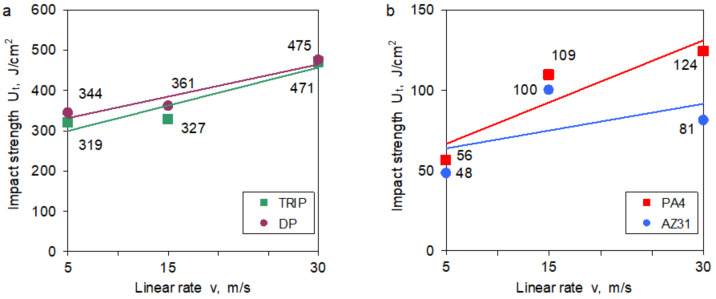
The impact strength as a function of the linear rate of the impact hammer for: (**a**) TRIP and DP steels and (**b**) PA4 and AZ31 alloys.

The results of the impact strength tests in the dynamic stretching tests showed an increase in the impact strength of TRIP and DP steels (Figure 10a). The nature of the curves is similar, slightly higher impact strength values were obtained for DP steel. Similarly, aluminum and magnesium alloys show an increase in impact strength across the entire strain rate range. A significant increase in the impact strength was demonstrated by the aluminum alloy PA4 (Figure 10b).

**Figure 11:**
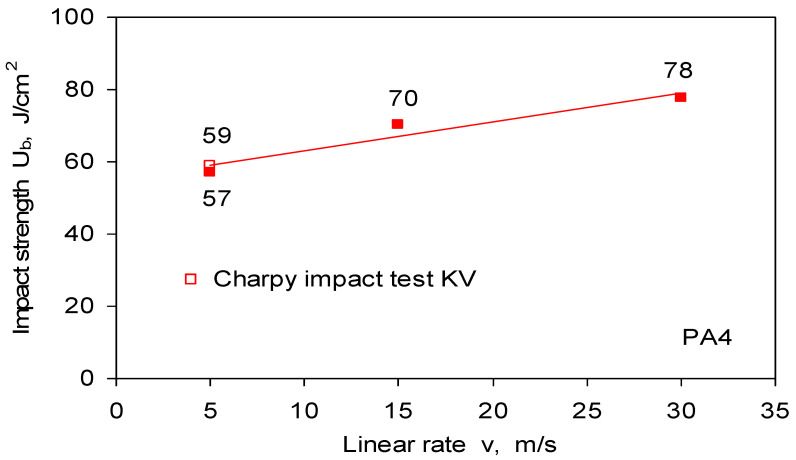
Toughness in tests of dynamic bending of PA4 aluminum alloy on rotational hammer. In the graph, for comparison, the impact result obtained on the Charpa pendulum was applied.

The performed impact bending tests on the rotary hammer of the PA4 aluminum alloy showed that the impact strength increases with the linear speed of the hammer and is greater than the impact strength determined in the standard bending test on the pendulum hammer (Figure 11). The linear speed of the Charpy hammer pendulum at the moment of breaking the sample is about 5 m/s. The impact strength results obtained on the rotary hammer and pendulum hammer for the linear speed of 5 m/s are similar and amount to 57 and 59 J/cm^2^, respectively. This proves the high sensitivity of the rotary hammer measuring system also in the initial range of the hammer’s linear speed and confirms the correctness of the system indications. 

## 4. Discussion

The tests of selected metallic materials showed significant changes in mechanical properties with an increase in the strain rate compared to those obtained in quasi-static conditions and in standard impact tests (Charpy hammer bending test). The results of the endurance tests were confirmed by microfractographic studies of the identification of the fractures on a scanning microscope. Attempting to stretch on a flywheel machine in a technically simple way gives this possibility, hence the results of microfractographic studies in publications. Compression tests on Hopkinson’s modified rods do not identify breakthroughs because the sample is not destroyed.

**Figure 12:**
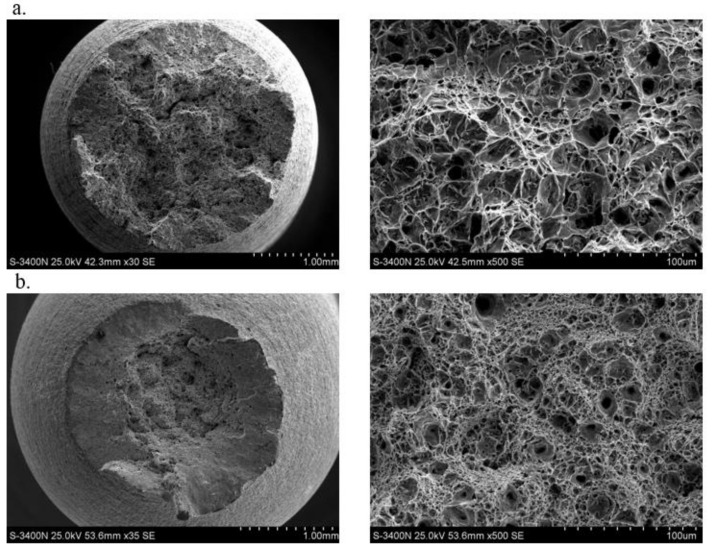
Specimen fractures after dynamic tension with the linear rate of the impact hammer v = 30 m/s for steels: (**a**) TRIP; (**b**) DP.

Microfractographic tests of TRIP and DP steels showed the ductile form of fractures of samples stretched under static conditions and dynamically deformed regardless of the linear speed of the hammer. In the morphology of fractures, pits characteristic for ductile cracking and cracks running through the grains along the cleavage planes were observed (Figure 12). A significant amount of carbide precipitation was observed in DP steel.

**Figure 13:**
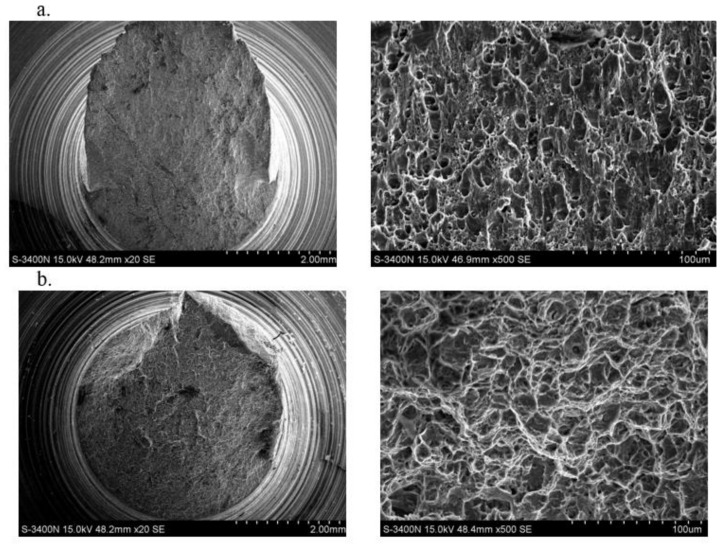
Specimen fractures after dynamic tension with the linear rate of the impact hammer v = 30 m/s for: (**a**) PA4; (**b**) AZ31.

In the nonferrous alloys PA4 and AZ31, the nature of the fractures was varied (Figure 13). In the PA4 aluminum alloy, there is a mixed fracture for all deformation rates. The areas of the brittle fracture are visible. Similarly, in magnesium alloy AZ31, the fracture in the ductile form dominates with areas of a brittle fracture character. 

TRIP and DP steels showed good plastic properties in the entire range of the linear speed of the hammer and a high tendency to intensively strengthen with an increase in the strain rate. This confirms the great usefulness of these construction materials in the construction of vehicle bodies, both in the technological process of manufacturing and during the operation of ready-made elements responsible for passive safety. In nonferrous alloys, in the morphology of fractures, the occurrence of areas of brittle fracture can be observed, which indicates the need for additional analyzes allowing the material to build dynamically loaded structural elements of vehicles and airplanes.

The conducted analysis of selected metallic materials does not exhaust the entire problem of the influence of high strain rates on mechanical and microstructural properties. However, it indicates the existence of a significant dependence between the dynamics of the deformation course and the mechanical properties and structural phenomena accompanying high rates of deformation.

There are few publications in the literature that present similar research results and discuss the possibilities of using the obtained databases in the structure design process [22]. This is undoubtedly due to limited access to unique research equipment, lack of experience or purely commercial reasons. The tests on the rotary hammer are competitive with the dynamic tests with the use of the Hopkinson bar. It is primarily the compact structure of the device, ease of testing, uncomplicated shape of the sample, low sensitivity of the measurement system to external interferences, simple way of processing the results and high repeatability. The test results can be directly applied to the design of both new construction materials and the analysis of the mechanisms of destruction of energy-consuming elements in technological tests carried out on drop hammers or entire objects in crash tests. The rates of deformation achieved in the deformation tests on the rotary hammer correspond to the values of the rates of deformation occurring locally in real objects subjected to extreme deformation conditions. This, of course, makes it possible to directly assess the behavior of the construction material already in the basic research phase without undertaking costly technological tests of elements or entire objects. The obtained characteristics of the course of deformation in the form of yield curves can be used as a database for the verification of the results of the FEM calculation [10,24] (or finite element analysis (FEA)).

## 5. Conclusions

The developed measuring system of the rotary hammer meets the requirements of recording quick and short-term signals of force and linear speed of the exciting element. The proposed research methodology is universal and applicable to a large group of metallic materials, from highly hardening modern structural steels used in the automotive industry, to nonferrous metal alloys commonly used in aircraft structures.

The number of dynamic deformation tests carried out on the bench in many research projects is very large and concerns a significant group of materials, steels and nonferrous metal alloys. Thus, statistical analyses of the results are also foreseen in order to determine a sufficient number of tests for the individual material to know the collective distribution in terms of the selected resulting characteristic and the type of relationship between factors, e.g., the linear speed of the forcing element and the strengthening of the material and structural changes. Currently, research is focused on improving the research and measurement technique—temperature measurement. The measurement of the sample temperature is essential for the correct microstructural assessment of the material due to the fact that the dynamic deformation takes place under adiabatic conditions, without replacing the flat with the environment.

The possibilities of using new construction materials in the automotive and aviation industries are more and more often determined by their resistance to shock loads, hence the need to develop new methods of testing materials under conditions of dynamic deformation and continuous improvement of measurement techniques. It is also necessary to develop a standard procedure that allows for an unequivocal evaluation of the results of dynamic deformation tests obtained in various kinematic conditions of the course of the experiment.

## Data Availability

The data presented in this study are available on request from the authors.
